# Quality of Life, Physical and Mental Health, and Economic Evaluation of Family Caregivers of Chronic Dependent Children: INFAPRINT Cohort Study Protocol

**DOI:** 10.3390/ijerph20065081

**Published:** 2023-03-14

**Authors:** Álvaro León-Campos, Silvia García-Mayor, Celia Martí-García, Juan Carlos Morilla-Herrera, José Miguel Morales-Asencio, Inmaculada Lupiáñez-Pérez, Bibiana Pérez-Ardanaz, Magdalena Cuevas Fernandez-Gallego

**Affiliations:** 1 Department of Nursing, Faculty of Health Sciences, University of Malaga, 29071 Malaga, Spain; 2 Institute of Biomedical Research in Malaga (IBIMA), 29590 Malaga, Spain; 3 Andalusian Health Service, District Malaga-Guadalhorce, 29004 Malaga, Spain

**Keywords:** nursing, caregivers, informal caregivers, paediatrics, quality of life, mental health, physical health, long-term care

## Abstract

Background: Caregivers for children with complex chronic illnesses may experience emotional and physical strain, especially as concerns attention overload and the perceptions of their own psychosocial situation. These concerns, together with the additional financial cost and the socioeconomic inequalities that arise from caregiving responsibilities, create major challenges to the health status of this population group. Methods: A prospective analytical longitudinal study will be conducted, based on an exposed cohort of adult caregivers (parents or guardians) for children with complex chronic processes, to evaluate the impact of caregiving responsibilities on the health status of this population group. Conclusions and implications: The practical implications of this study are of great significance for clinical practice. The results of this study have the potential to inform the decision-making process in the healthcare sector and guide future research initiatives. The findings of this study will provide crucial insights into the health-related quality of life of caregivers of children with complex chronic illnesses, which will be valuable in addressing the challenges faced by this population group. This information can be used to improve the availability and accessibility of appropriate health services and to facilitate the development of more equitable health outcomes for caregivers of children with complex chronic illnesses. By highlighting the extent to which this population is affected both physically and mentally, the study can contribute to the development of clinical practices that prioritize the health and well-being of caregivers in the care of children with complex chronic illnesses.

## 1. Introduction

Chronic illness was defined as a condition that interferes, or is likely to interfere, with a person’s daily functioning for at least three months in a year, or a condition requiring hospitalization for more than one month in a year [1].

The UK National Institute of Health and Care Excellence (NICE) [2] defined multimorbidity as the concurrent presence of multiple conditions that produce significant functional deterioration in the activities of daily life, or as a situation in which health care provoked an overload or involves a high level of need for unrelated health services. In Spain, 19.49% of children aged under 14 years were reported to suffer from a chronic pathology. In Andalusia (southern Spain), this prevalence was reported to be even higher, at 20.62%, according to the 2017 National Health Survey [3]. The most prevalent chronic diseases among children were reported to be allergies (11.43%), asthma (3.84%) and behavioural disorders (2.53%). Family life may be disrupted in many ways following the diagnosis of a serious illness, and during this period, children are exposed to significant levels of psychosocial stress [4].

The presence of disease in a family member can bring about significant changes in the daily life and routine of the household, leading to the need for both psychological and practical adjustments. This can result in a heightened risk for the entire family structure of developing psychological distress, including symptoms of acute stress disorder [5,6]. Stress is a non-specific bodily response to excessive environmental demands. When a child has a chronic illness, the family may experience significant attention overload, which increases the demands placed on it and may require a reorganization of roles [7]. In addition, the way in which each parent perceives and reacts to their psychosocial situation can affect the parent–child relationship and the child’s development [8].

In such situations, parents may encounter heightened levels of anxiety due to the uncertainty surrounding the most effective means of supporting their children. However, despite this, they frequently receive little to no assistance or guidance in addressing this delicate matter [9]. A study conducted in the USA [10] on the health-related quality of life (HRQoL) of parents of children with epilepsy, parents of children with chronic diseases and parents of healthy children showed that the HRQoL of parents of children with chronic diseases was poorer, emotionally, physically and socially, than that of parents of healthy children. Additionally, there is a perceived gender disparity in caregiving, particularly in cases where the child has a chronic illness. Recent studies [11,12] on caregiving as part of the management of chronic diseases in children and adolescents reported that it is usually the mother who plays the main role in this respect, in addition to domestic work and other parenting responsibilities. Thus, caring for children with chronic illnesses is a complex and demanding undertaking [13].

As well as the emotional and physical overload related to the care of dependent children, this type of informal care produces additional financial costs. Socioeconomic inequality is known to be a key determinant of health, in people of all ages, and presents major challenges for medical care worldwide [14]. A down-sloping socioeconomic gradient is associated with a corresponding decline in health outcomes among school-age children [15]. Children and adolescents from socioeconomically underprivileged families are more than twice as likely to experience acute illness or chronic illnesses such as asthma, obesity [16], mental illness [17] or developmental delay [18], compared to those from a wealthier background. However, while caregiver burden in general is well documented, little is known about the particular impact placed on those who care for a dependent child.

In response, there is growing research interest in determining the secondary impact of children’s illness on caregivers and other family members, and in incorporating these broader effects into economic evaluations of health. In this respect, studies indicated that these effects of the disease can be measured, but tend to be of small magnitude and vary according to the child’s condition and the population affected [19]. These inferences are based on limited research, but increasing attention is being paid to this problem [20,21]. It is necessary to accurately measure the resources consumed in the paediatric response to the disease, considering the effect size, which can be determined using health service weighting criteria [22]. In view of the considerable impact of chronic disease, it is important to characterise the variability in these results in order to identify associations in which secondary impacts of disease should be included in healthcare-related economic assessments.

In practice, however, the full scope of caregiver effects is not usually included in cost-effectiveness analysis. While the direct costs associated with time spent in informal care are normally included, the reduced quality of life of the informal caregiver (or other family members) is often overlooked. This omission may be attributable, in part, to the inherent difficulty of measuring indirect consequences on the caregiver’s quality of life.

In view of these considerations, our study aims to address two fundamental questions related to the care of children with chronic illnesses: firstly, we consider whether there is a significant difference in the HRQoL of parents/legal guardians who care for children with complex chronic illnesses, compared with informal caregivers of healthy children, and whether any such difference might generate health inequalities. Secondly, we consider how socioeconomic factors (such as the caregivers’ occupation, social class and income) might be related to the HRQoL, both that of the caregivers and that of children with complex chronic illnesses.

### 1.1. Aims

#### 1.1.1. General Aims

To determine the impact of family caregiving for children with complex chronic illness on the mental health of their caregivers, compared to caregivers of healthy children.

To analyse the association between family caregivers’ exposure to child care responsibilities, on the one hand, and their anxiety, depression and perceived reduced quality of life (physical and mental health) on the other.

#### 1.1.2. Specific Aims

1. To analyse and identify the associations between social class, family income and HRQoL of children with common childhood chronic diseases;
2. To determine the relationship between the HRQoL of family caregivers and that of their children;
3. To analyse the association between the family care of a child and the use made of healthcare services (primary and/or specialised);
4. To identify the characteristics of these caregivers and of the care provision associated with improving or worsening perceptions of physical and mental health, taking into account changes in the care burden and in the number of hours dedicated to care.

## 2. Materials and Methods

Analytical longitudinal study of prospective cohorts (cohort study).

### 2.1. Setting

Primary healthcare districts of Málaga-Valle del Guadalhorce (southern Spain).

### 2.2. Participants

The exposed cohort will be composed of family caregivers (parents or legal guardians) of children aged between 6 months and 16 years, with complex chronic disease, according to the NICE criteria [2]: children with chronic conditions who:

- have difficulty managing their treatment or performing normal activities of daily living;
- receive care and support from multiple health services;
- present chronic physical and/or mental health problems;
- frequently require unplanned or emergency care;
- are prescribed multiple medications.

In addition, the following criteria regarding the theoretical framework for children with medical complexity, presented by Cohen [23], will be applied:

- children with chronic conditions who frequently need healthcare services;
- children whose activities of daily life are subject to significant limitations.

With these criteria in mind, the exposed cohort will be classified into four groups, in accordance with the method adopted in the Feudtner study [24], and coded using the ICD-10 classification system [25] (Table 1).

The non-exposed cohort will comprise family caregivers of children aged 6 months to 16 years who do not have complex chronic processes (although they may present chronic diseases if these do not meet the Feudtner criteria and those of chronic care management, CCM), or who are completely healthy.

The participants will be identified from DIRAYA, the digital health record system of the Andalusian public health service, in accordance with the above criteria.

### 2.3. Inclusion Criteria for Caregivers

Caregivers (parents or legal guardians), aged over 18 years, of a child with a complex chronic process (exposed group) according to Feudtner, NICE and CCM criteria, during the study period. Informed consent will be requested of all participants. Whenever possible, information will be collected from all caregivers and parents.

### 2.4. Exclusion Criteria

Minors whose life expectancy does not exceed the follow-up time required to complete the study (12 months) or who do not have a clear diagnosis of chronic disease will be excluded. In addition, any caregiver who presents cognitive impairment or intellectual disability or whose command of Spanish is insufficient for them to complete the questionnaires and information collection instruments will be excluded from the study.

### 2.5. Data Collection

#### Recruitment and Monitoring of Exposed and Non-Exposed Participants

Study participants will be recruited after consulting digital healthcare records referring to chronic patients and by means of non-probabilistic sampling by quotas, ensuring the adequate representation of all age strata and degrees of dependence.

The Spanish-language versions of the clinimetric tools to be used were all validated (see the bibliographic references in this respect) and can be administered by telephone, to safeguard the individuals’ health in the current pandemic situation.

Follow-up: Study participants will be recruited (by telephone) by the technical staff hired for this purpose. Follow-up interviews will take place every six months during the 18-month follow-up period, when the study variables will be reassessed. All data will be anonymously entered into a database, with the Andalusian Health system ID as the only identification.

A 2019 systematic review [26], comparing the anxiety and depression experienced by caregivers for children with complex chronic illness and by parents of healthy children reported a standardised mean difference of 0.42 [0.24–0.60] for anxiety and 0.35 [0.26–0.45] for depression. In other words, the caregivers of children with complex chronic diseases presented poorer mental health. However, both of these meta-analyses are subject to high levels of heterogeneity (I^2^ > 50%).

From these findings, and assuming a 1:1 sample size ratio and a confidence level of 95%, we calculated that the sample size for inter-group comparison should be 90 individuals in each group (exposed and unexposed) for anxiety and 130 for depression.

Using the largest of the sample calculations (the depression variable), the statistical power obtained would be sufficient to corroborate the study hypothesis for all the dimensions studied (HRQoL, anxiety and depression). The sample of complex chronic children will be divided into four groups, in accordance with Feudtner’s criteria [24], and, therefore, the final sample size will be multiplied by four, to compensate for the increased probability of type I error in the comparisons. In addition, the total sample will be increased by 20% to counteract possible losses to follow-up. Therefore, the sample will consist of 624 caregivers in the exposed group and 156 in the unexposed group, thus making 780 individuals in total.

### 2.6. Variables

#### 2.6.1. Sociodemographic Variables

Sociodemographic data on both the caregiver and child with chronic disease will be collected. This will include continuous quantitative variables such as age and duration of chronic process in months. Dichotomous qualitative variables such as sex for both the child and caregiver will also be collected.

Additionally, qualitative polychotomous variables such as caregiver’s occupation, education background and marital status will be gathered. The child’s level of dependency will be assessed based on their chronic process suffered and score on a dependency assessment scale (BVD) for children aged 3 to 17 years. The degree of dependency in activities of daily living will be measured by the BVD. For those aged under 3 years, a specific assessment scale (EVE) will be used.

Furthermore, the level of family support will be evaluated using the family APGAR index [27]. Family function will be measured by this tool by assessing five dimensions: adaptation, partnership, growth, affection and resolve.

#### 2.6.2. Economic Variables

Economic data including monthly family income (self-declared), assistance received under the Dependency Law (qualitative dichotomous variable), amount of Dependency Law benefit received (qualitative polychotomous variable) will also be collected, which is only relevant to the exposed group. The Dependency Law (Ley de Dependencia) is a law in Spain that provides access to many services and benefits funded partially or totally by the State for dependent people of all ages. Public support for people who cannot lead independent lives for reasons of illness, disability or age is guaranteed by it. To access these services, an application for the “Dependency Law” has to be made through their local Spanish Social Services department by the dependant or their representative.

Additionally, daily time spent caregiving in hours and duration of care in months (polychotomous qualitative variables) will be recorded for exposed group only.

Social class will be measured using the classification proposed by the Spanish Society of Epidemiology [28], based on occupation.

Regarding cost of care, we will measure both direct and indirect costs associated with caregiving. Direct costs will include the use of healthcare services for both the child and caregiver. This will include hospital admissions during follow-up, visits to the Emergency Department during the same period, visits to the family doctor, visits to the family nurse and visits to hospital specialists (discrete quantitative variables).

We will also consider technical assistance such as hiring formal support services, home reforms to meet the child’s needs, consultations with private health services and diagnostic tests. The costs of these health resources will be estimated according to public prices published by the Andalusian Public Health System [29]. The cost of direct resources related to intervention will be estimated in accordance with their official prices.

Indirect costs will include giving up paid employment to fulfil caregiving responsibilities, days of absence from work due to caregiving responsibilities, distance between home and primary healthcare centre (transport), daily care time spent on caregiving responsibilities and expenditure on special diet according to the child’s pathology. 

#### 2.6.3. Quality of Life and Mental Health

The mental health of caregivers of children with chronic diseases will be evaluated using several variables. Depression will be assessed using the Patient Health Questionnaire-9 (PHQ-9) [30], a self-report tool that measures the severity of depressive symptoms. Anxiety will be measured using the Generalized Anxiety Disorder-7 (GAD-7) [31], a self-report questionnaire that screens for generalized anxiety disorder.

The quality of life of the care recipient will be evaluated using the age-appropriate version of the Paediatric Quality of Life Inventory (PedsQL) [32]. This instrument assesses five domains of health in children and adolescents of ages 2 to 18: physical functioning, emotional functioning, social functioning, school functioning and psychosocial health. The caregiver’s quality life will be assessed using the Short Form Health Survey (SF-12) [33], a widely used measure that assesses physical and mental health.

Overload will also be measured as a discrete quantitative variable using the Caregiver Strain Index (CSI) [34]. This tool can identify strain and areas where support may be needed by assessing 13 items related to time demands, emotional adjustment, role strain and personal strain.

Consumption of psychotropic drugs will also be recorded via electronic prescription through the Andalusian electronic medical record system.

Additionally, information on threatening experiences will also be collected through a structured interview using the List of Threatening Experiences scale (LTE) [35]. This scale measures exposure to stressful events over a specified period. 

### 2.7. Data Analysis

All analyses will be carried out a blinded evaluator, working independently of the research team.

Descriptive and exploratory analysis: descriptive statistics of the variables will be determined, obtaining measures of central tendency and dispersion or percentages, depending on their nature. In every case, the normality of the distribution will be evaluated, using the Kolmogorov–Smirnov test, and tests of histograms, asymmetry and kurtosis.

Bivariate analysis: the chi-square test will be performed and the Mantel–Haenszel statistics obtained, applying Fisher’s exact correction, if necessary, for the qualitative variables. For all parameters, precision will be estimated according to the corresponding 95% confidence intervals. For normally distributed continuous variables, bivariate analysis will be performed using Student’s *t*-test for independent samples. If the distribution is non-normal, the Mann–Whitney U and the Wilcoxon non-parametric tests will be used. Inter-group analyses will be performed using ANOVA (after searching for homogeneity of variances by Levene’s test), together with post hoc analysis by Bonferroni’s test or by Welch and Hosmer–Lemeshow robust tests if non-homoscedasticity is detected. If these calculations cannot be performed due to the non-normality of the distributions, the Kruskall–Wallis test will be used instead. Finally, Dunnett’s test will be used to compare subgroups of childhood chronicity with the unexposed group (healthy children).

The sample will be stratified according to the differential values of the main variables (mental health, strain, quality of life and use of healthcare services) and the sociodemographic determinants (age and level of dependency of the care recipient) will be considered.

Multivariate analysis: multivariate analysis will be performed using logistic or multinomial regression, according to the variable analysed, to determine factors associated with the variables of interest. For this purpose, predictor variables will be assumed to be those with a significant association, according to the bivariate analysis. Those related to the main study goals (direct and indirect costs, use made of healthcare services, HRQoL and physical and mental health) will be taken as dependent variables, via models constructed to identify baseline differences. Possible confounding or effect modification effects will be explored and taken into account to adjust subsequent calculations. The longitudinal analysis will include Cox proportional models, dichotomising the HRQoL variable from a cut-off point to generate the outcome “poor quality of life”, as a relevant factor in calculating the hazard ratio. The Klein-Geltink et al. criteria for data analysis will be used to control for confounding factors.

## 3. Discussion

Parents may experience significant stress and hardship upon receiving the diagnosis of their child with a chronic illness. This often involves grappling with the medical risks associated with the condition and, for some, the prospect of a reduced life expectancy for their child. Despite some families exhibiting resilience in the face of such challenges, the demanding treatment regimen and the changes in roles, responsibilities and resources can have a detrimental impact on family functioning [36]. The results of this review indicate that economic difficulty, the nature and severity of treatment received by the child, and socio-economic class play crucial roles in determining the physical, psychological and social impact on family functioning caused by a child’s chronic illness [37]. Parents of children with chronic illnesses require solutions to problems, information and financial and practical help, especially when their children suffer from disabilities, cognitive impairment and/or social dysfunction. A multidisciplinary approach is essential to enable these parents to improve their skills and results. Most caregivers believe they are inadequately equipped for the tasks they need to perform, and have never received any formal training in caregiving [38]. In addition, most believe they need more information about support services [39,40]. Educational interventions in this respect can improve clinical outcomes, the care provided and the quality of life for caregivers of children with chronic illnesses.

To our knowledge, no studies have yet been conducted in Spain on the economic impact arising from the care of children with chronic diseases. This question was not even addressed in the National Health Survey, which compiles data on dependency for all age ranges, including children. This lack of information (in areas such as caregivers’ profile and specific difficulties) greatly hinders the application of measures targeting this population group.

Few studies were undertaken to characterise and reduce health inequalities among children, with most research in this area being aimed at the prevention of childhood diseases. Therefore, further efforts are required to gain a deeper understanding of the circumstances surrounding children with complex chronic diseases, in order to aid in the development of multidisciplinary strategies aimed at enhancing their health-related quality of life and comprehending the progression of the illness. Among other outcomes, this would help reduce the number of unscheduled visits made to primary and specialised healthcare centres.

Despite the limited evidence available, the literature review carried out as part of this project suggested that interventions to strengthen relationships with health services and improve accessibility are among the most important needs for caregivers. Advances in these respects would enhance the HRQoL of children with complex chronic disease and that of their caregivers. Finally, we hope the present study may constitute a solid basis for further high-quality systematic reviews facilitating the design and application of more accessible health management policies for this vulnerable population group.

## 4. Limitations

Due to the observational nature of this study, causalities between the factors analysed cannot be determined, although the longitudinal design and the specific characteristics of the study may create the necessary conditions to generate testable hypotheses in future work. The ‘social class’ variable cannot be used for all caregivers because the standardised classifications currently available do not include the unemployed and persons aged over 65 years, a shortcoming that could result in the omission of significant numbers of persons who meet the criteria for inclusion in the study. Similarly, the ‘income level’ variable is subject to the limitation that access to objective data, in this respect, may be lacking. If it is unavailable, this information (referring to income before receiving any benefit or aid) will be requested from the caregivers, at the beginning of the study. Nevertheless, we realise the information provided may contain biases due to potential conflicts of interest with the receipt of such benefits and aid. However, other variables will be taken into account, such as the caregivers’ education background and occupation.

## 5. Conclusions

The findings from this study will have several implications for clinical practice. Firstly, it highlights the importance of evaluating the mental health of caregivers of children with complex chronic illnesses, as they may experience increased levels of anxiety and depression compared to caregivers of healthy children. This highlights the need for mental health support for these caregivers.

Additionally, the study’s analysis of the relationship between social class, family income and the HRQoL of children with common childhood chronic diseases highlights the need to consider the socio-economic status of families when providing care.

Furthermore, the determination of the relationship between the HRQoL of family caregivers and their children has important implications for the assessment of family functioning and the provision of support to families.

Lastly, the analysis of the association between the practice of family care and the use of healthcare services (primary and/or specialised) highlights the need for integrated care that considers the family context when providing care for children with chronic illnesses. This study underscores the need for healthcare providers to be attentive to the characteristics of caregivers and the care provision process in order to improve the physical and mental health of both the caregivers and the children in their care.

By determining the extent to which caregivers of children with complex chronic illnesses are affected, physically and mentally, and identifying the socioeconomic and HRQoL impact of caring for these children, we hope to foster the development of more equitable health outcomes for this population, by improving the availability, accessibility and appropriateness of the health services provided to this population group and by developing policies tailored to this specialised area of paediatric care.

## Figures and Tables

**Table 1:** Group classification according to Feudtner criteria and CIE-10.

	Examples	CIE-10 Codes
Group 1		
Conditions that are life threatening or those with feasible treatment but that with possibility of failure.	Cancer, irreversible organic failure: heart, liver, kidney.	G89.X
Group 2		
Conditions where premature death is unavoidable, or where there may be long periods of intensive treatment with the goal of prolonging life and allowing participation in normal activities.	Severe immunodeficiencies, renal failure without the possibility of transplantation or dialysis. Severe or chronic respiratory failure (cystic fibrosis, HIV, muscular dystrophy).	D80-D89, N17-N19, T82.41, Z71.7, Z13.228, GT71.0
Group 3		
Progressive conditions without curative treatment options, or where palliative treatment may extend for several years.	Mucopolysaccharosis and other severe metabolic diseases, severe chromosomal disorders, neuromuscular diseases, severe osteogenesis imperfecta (Batten disease, spinal muscular atrophy, trisomy 13 or trisomy 18, severe infant suffocation)	E00-E89, Q00-Q99, G70.X, E75.X
Group 4		
Irreversible, but not progressive, conditions that cause severe disabilities leading to susceptibility to health complications and the likelihood of premature death.	Severe cerebral palsy, multiple disabilities, such as after a brain or spinal cord injury, complex health care needs, and a high risk of an unpredictable and life-threatening event or episode. Severe neurological diseases, severe brain malformations, hypoxic brain damage.	F06.X, I63.X, I67.X, Q28.X

## Data Availability

Data sharing is not applicable to this article as no datasets were generated or analysed yet.

## References

[1] OMS Enfermedades No Transmisibles. Who. 2017. p. 1. https://www.who.int/es/news-room/fact-sheets/detail/noncommunicable-diseases.

[2] Kernick D., Chew-Graham C.A., O’Flynn N. (2017). Clinical assessment and management of multimorbidity: NICE guideline. Br. J. Gen. Pract..

[3] Problemas o Enfermedades Crónicas o de Larga Evolución en los Últimos 12 Meses en Población Infantil Según Sexo y Grupo de Edad. Población de 0 a 14 Años. https://www.ine.es/jaxi/Tabla.htm?path=/t15/p419/a2017/p04/l0/&file=02024.px&L=0.

[4] Fearnley R., Boland J.W. (2017). Communication and support from health-care professionals to families, with dependent children, following the diagnosis of parental life-limiting illness: A systematic review. Palliat. Med..

[5] Werner-Lin A., Biank N.M. (2009). Along the Cancer Continuum: Integrating Therapeutic Support and Bereavement Groups for Children and Teens of Terminally Ill Cancer Patients. J. Fam. Soc. Work..

[6] Ernst J., Götze H., Brähler E., Körner A., Hinz A. (2012). Quality of life of parents diagnosed with cancer: Change over time and influencing factors. Eur. J. Cancer Care.

[7] de Paula É.S., Nascimento L.C., Rocha S.M. (2008). Role assessment in families of children with chronic renal failure on peritoneal dialysis. Int. J. Nurs. Pract..

[8] Morrow A.M., Hayen A., Quine S., Scheinberg A., Craig J.C. (2012). A comparison of doctors’, parents’ and children’s reports of health states and health-related quality of life in children with chronic conditions. Child Care Health Dev..

[9] Turner J., Clavarino A., Yates P., Connors V., Hargraves M., Hausmann S. (2007). Development of a resource for parents with advanced cancer: What do parents want?. Palliat. Support Care.

[10] Haneef Z., Grant M.L., Valencia I., Hobdell E.F., Kothare S.V., Legido A., Khurana D. (2010). Correlation between child and parental perceptions of health-related quality of life measurement model. Epileptic Disord..

[11] Flórez-Torres I.E., Montalvo-Prieto A., Herrera-Lían A., Romero-Massa E. (2011). Afectación de los bienestares en cuidadores de niños y adultos con enfermedad crónica. Rev. Salud Pública.

[12] Rehm S.R. (2013). Nursing’s contribution to research about parenting children with complex chronic conditions: An integrative review, 2002 to 2012. Nurs. Outlook.

[13] Macedo E.C., Da Silva L.R., Paiva M.S., Ramos M.N.P. (2015). Burden and quality of life of mothers of children and adolescents with chronic illnesses: An integrative review 1. Rev. Lat. Am. Enferm..

[14] Didsbury M.S., Kim S., Medway M.M., Tong A., McTaggart S.J., Walker A.M., White S., Mackie F.E., Kara T., Craig J.C. (2016). Socio-economic status and quality of life in children with chronic disease: A systematic review. J. Paediatr. Child Health.

[15] Fernández D.L.H.K., Leon D.A. (1996). Self-perceived health status and inequalities in use of health services in Spain. Int. J. Epidemiol..

[16] Ruijsbroek A., Wijga A.H., Kerkhof M., Koppelman G.H., Smit H.A., Droomers M. (2011). The development of socio-economic health differences in childhood: Results of the Dutch longitudinal PIAMA birth cohort. BMC Public Health.

[17] Goodman E. (1999). The role of socioeconomic status gradients in explaining differences in US adolescents’ health. Am. J. Public Health.

[18] Najman J., Bor W., Morrison J., Andersen M., Williams G. (2002). Child developmental delay and socio-economic disadvantage in Australia: A longitudinal study. Soc. Sci. Med..

[19] Wittenberg E., Prosser L.A. (2013). Disutility of illness for caregivers and families: A systematic review of the literature. Pharmacoeconomics.

[20] Bobinac A., van Exel N.J.A., Rutten F.F.H., Brouwer W.B.F. (2010). Caring for and caring about: Disentangling the caregiver effect and the family effect. J. Health Econ..

[21] Wittenberg E., Ritter G.A., Prosser L.A. (2013). Evidence of spillover of illness among household members: EQ-5D scores from a US sample. Med. Decis. Mak..

[22] Lavelle T.A., Wittenberg E., Lamarand K., Prosser L.A. (2014). Variation in the Spillover Effects of Illness on Parents, Spouses, and Children of the Chronically Ill. Appl. Health Econ. Health Policy.

[23] Cohen E., Kuo D.Z., Agrawal R., Berry J.G., Bhagat S.K.M., Simon T.D., Srivastava R. (2011). Children with Medical Complexity: An Emerging Population for Clinical and Research Initiatives. Pediatrics.

[24] Feudtner C., Feinstein J.A., Satchell M., Zhao H., Kang T.I. (2007). Shifting Place of Death Among Children with Complex Chronic Conditions in the United States, 1989–2003. JAMA.

[25] Ministerio de Sanidad, Servicios Sociales e Igualdad (2018). CIE-10-ES: Clasificación Internacional de Enfermedades–10.Œ Revisión: Modificación Clínica.

[26] Cohn L.N., Pechlivanoglou P., Lee Y., Mahant S., Orkin J., Marson A., Cohen E. (2020). Health Outcomes of Parents of Children with Chronic Illness: A Systematic Review and Meta-Analysis. J. Pediatr..

[27] Smilkstein G. (1978). The Family APGAR: A Proposal for a Family Function Test and Its Use by Physicians. J. Fam. Pract..

[28] Domingo-Salvany A., Regidor E., Alonso J., Álvarez-Dardet C. (2000). Proposal for a social class measure. Working Group of the Spanish Society of Epidemiology and the Spanish Society of Family and Community Medicine. Aten. Primaria.

[29] Junta de Andalucía (2020). Junta de Andalucía—Datos Abiertos: Catálogo de Datos. https://www.juntadeandalucia.es/datosabiertos/portal/dataset/precios-publicos-de-servicios-sanitarios-prestados-en-el-sspa.

[30] Diez-Quevedo C., Rangil T., Sanchez-Planell L., Kroenke K., Spitzer R.L. (2001). Validation and Utility of the Patient Health Questionnaire in Diagnosing Mental Disorders in 1003 General Hospital Spanish Inpatients. Psychosom. Med..

[31] Garcia-Campayo J., Zamorano E., Ruiz M.A., Pardo A., Perez-Paramo M., Lopez-Gomez V., Freire O., Rejas J. (2010). Cultural adaptation into Spanish of the generalized anxiety disorder-7 (GAD-7) scale as a screening tool. Health Qual. Life Outcomes.

[32] Girabent-Farrés M., Bagur-Calafat C., Amor-Barbosa M., Natera-De Benito D., Medina-Rincón A., Fagoaga J. (2019). Spanish Translation and Validation of the Neuromuscular Module of the Pediatric Quality of Life Inventory (PedsQL): Evaluation of the Quality of Life Perceived by 5–7 Years Old Children with Neuromuscular Disorders and by Their Parents. Rev. Neurol..

[33] Vilagut G., Ferrer M., Rajmil L., Rebollo P., Permanyer-Miralda G., Quintana J.M., Alonso J. (2005). The Spanish Version of the Short Form 36 Health Survey: A Decade of Experience and New Developments. Gac. Sanit..

[34] Robinson B.C. (1983). Validation of a Caregiver Strain Index. J. Gerontol..

[35] Motrico E., Rodero-Cosano M.L., Álvarez-Gálvez J., Salinas-Pérez J.A., Moreno-Peral P. (2017). Instrumentos de evaluación de los eventos vitales estresantes en población española adulta: Una revisión sistemática. An. Psicol. Univ. Murcia Serv. Publ..

[36] Cousino M.K., Hazen R.A. (2013). Parenting Stress Among Caregivers of Children with Chronic Illness: A Systematic Review. J. Pediatr. Psychol..

[37] Darwish M.M., Hassan S.H., Taha S.F., Abd El-Megeed H.S., Ismail T.A.A.M. (2020). Family impact and economic burden among caregivers of children with chronic kidney disease in Assiut, Egypt. J. Egypt Public Health Assoc..

[38] Berry J.G., Hall M., Neff J., Goodman D., Cohen E., Agrawal R., Kuo D., Feudtner C., Colvin J.D., Richardson T. (2014). Children WITH Medical Complexity And Medicaid: Spending And Cost Savings. Health Aff..

[39] Foster C.C., Shaunfield S., Black L.E., Labellarte P.Z., Davis M.M. (2021). Improving support for care at home: Parental needs and preferences when caring for children with medical complexity. J. Pediatr. Health Care.

[40] Masaba B.B., Taiswa J., Mmusi-Phetoe R.M. (2021). Challenges of Caregivers Having Children with Autism in Kenya: Systematic Review. Iran. J. Nurs. Midwifery Res..

